# A Treatise on Neuralgia

**Published:** 1839-01-01

**Authors:** 


A Treatise on Neuralgia. By *Richard Rowland*, M.D. Physician to the City Dispensary. 8vo. pp. 174. Highley, Sept. 1838.

Neuralgia is a rapidly increasing malady; and consequently we may expect successive publications on the subject. The torture occasioned by the disease—its uncertain and treacherous attacks—the almost impenetrable obscurity in which it is shrouded—and lastly the difficulty of cure, must always render neuralgia an interesting subject of inquiry, both amongst physicians and the sufferers themselves. There is no doubt, too, but that young practitioners every day confound neuralgia with phlogosis, by which much mischief is occasioned. There is no internal organ or structure of the body exempt from attacks of neuralgia, and it may be very easily believed that when such parts as the heart, the pleura, the peritoneum, &c. are invaded, the inexperienced, and still more the incautious and routine practitioner, may be readily thrown off his guard, and unsheath his lancet against the formidable enemy in the shape of carditis, pleuritis, peritonitis, &c. Even when neuralgia exhibits the chief characteristics of inflammation, rubor, tumor, dolor, calor, as is sometimes seen, the inflammation is of a peculiar kind that ill bears depletion of the vascular system. Who has not seen, in "brow ague," as it is called, the conjunctiva, the palpebræ, and, in short, all the visible structures of the eye and its connexions, become scarlet, hot, swelled, and painful, thus fulfilling the four essential requisites of phlogosis—yet all these phenomena will disperse in a few hours, without leeches, lotions, or purgatives.

Phlogosis is not nearly so often mistaken for neuralgia, as the latter for the former. It is so far fortunate; for to treat inflammation as a neurosis would be fatal.

Dr. Rowland's work is a regular systematic treatise, compiled from all accessible authorities, and containing scarcely anything original, except a few cases at the end. It would therefore make a capital article (somewhat condensed) for an enlarged cyclopædia of medicine. We shall not attempt an analysis. We might as well try to analyze a pocket dictionary. Dr. R.'s book is a very useful one, as collecting into a focus the rays of light—or at least of supposed light—scattered through multitudinous journals, memoirs, and monographs. It might not be either useless or uninteresting to present our readers with a kind of synopsis of the various *causes* that have been assigned for neuralgia—the conjectures as to its seat and nature—and the remedies that have been proposed for its cure or mitigation.

### I. Exciting Causes.

1. Exposure to *wet and cold* is one of the most common exciting causes. 2. *Cutaneous irritation,* as contusions, eruptions, cicatrices, &c. Herpes zoster is accompanied by so much pain as to resemble neuralgia. 3. *Tension* of nerves. The stretching of limbs, the suspension of heavy weights, violent twistings and contortions of the trunk or members have excited neuralgia. 4. *Pressure* on nerves, as from a foreign body, enlarged organ, dilated vessel, tumour, &c. The subcutaneous tubercle is attended by an almost neuralgic pain. 5. *Carious teeth.* This is a very common cause of facial neuralgia. 6. *Disorder of the alimentary canal.* Great diversity of opinion prevails on this point—very many attributing neuralgia to chylopoietic derangement where no other cause is evident, while some distinguished practitioners deny the reality of this cause altogether. Among the latter, for example, are Montfalcon and Dr. Elliotson. Most of the observant and unprejudiced practitioners will be inclined to believe that irritation of the gastro-intestinal nerves is very frequently the cause of painful states of other nerves. The late Dr. Wollaston was stricken with neuralgia by eating an ice-cream. He became sick, threw up the ice-cream, and the pain disappeared. Sir B. Brodie relates that and some other similar cases. 7. Diseases of the *urinary organs.* Sir B. Brodie and others have related cases where severe neuralgia was produced by this class of causes. 8. Disorders of the *heart and large vessels.* We have some doubts of this source of neuralgia, except where enlargements act mechanically on the adjacent nerves. 9. *Uterine disorders.* This source has been well attested by numerous and competent witnesses. This may account for the greater frequency of neuralgia in women than in men—and also for the circumstance that it occurs not unfrequently about the approach of the catamenia. "That peculiar form of neuralgia which simulates visceral inflammation, very commonly originates in uterine disorder." 10. *Spinal irritation.* This source of the complaint in question, was pointed out so long ago as 1785, by

Pouteau, and more recently by Bradley, Brown (Glasgow), Darwall (Birmingham), Teale (Leeds), and others. In these cases, pain is seldom complained of in the back itself, except when examined by pressure or hot sponge. Then the patient shrinks when a particular spot is touched. There is a very general relation between the seat of the neuralgic pain and the portion of spinal column where the tenderness is felt, readily enough traced by the anatomist. It is to be remembered, however, that spinal irritation may itself be caused by disorder in other parts, as in the line of the digestive organs, and then the two affections act and re-act on each other. 11. *Organic disease of the brain and spinal marrow.* This is not confined solely to the cerebral nerves, but extends to nerves of spinal origin—or even of the ganglionic class, as those of the stomach, liver, or other internal organs, whilst the cerebral symptoms are absent or very slight. Thus Andral relates a case where ramollissement was going on in the brain, but the only symptom was pain in the lower extremities. A patient in Bartholomew's Hospital had such violent pain in the knee that the limb was amputated, but nothing found to account for the pain. Some years afterwards the patient died, and then plates of cartilaginous and bony deposits were found in the posterior surface of the spinal cord. 12. *Malignant Diseases.* The dreadful lancinating pains attending malignant diseases seem to be neuralgic. 13. *Chronic inflammation.* The pains in these cases, are generally periodical, and very different from those of ordinary chronic phlogosis. 14. *Malaria.* Van Swieten observed the striking analogy between ague and periodical neuralgia; but it was Macculloch, as our readers know, who traced, with a masterly hand, neuralgia to malaria. Scarcely a day passes that we do not observe neuralgia clearly traceable to this cause, especially among those who are mad enough to spend the Summer in Italy and other malarious countries.

### II. Seat of Neuralgia.

Hardly any one will doubt that the seat of this disease is in the nerves. It was long doubted, and even denied, that the ganglionic nerves could be the seat of pain, as vivisections had shewn that they might be pricked or cut without sensation. Most physiologists—especially those who are in active practice—now admit that the sensibility of the ganglionic nerves may be raised by disease to that degree possessed by the cerebro-spinal nerves. Brachet has set this matter in a clear point of view.

" He found that the sympathetic ganglia and the filaments which proceed from them, might be repeatedly pricked, without any sign of suffering being shewn by the animal, but when the irritation was continued until the ganglia became red and inflamed, that acute pain was then produced by every puncture; that when a ganglion had been thus irritated, and rendered sensible, its sensibility was again destroyed by the section of the nerve which connected it with the spinal marrow; but if the irritation were renewed after the lapse of a few minutes, the ganglion was found to have regained its sensibility, of which, however, it was finally and completely deprived by making a section of the nerves of communication passing between it and the ganglia situated immediately above and below it; that when a ganglion had been excited to sensibility, subsequently to the section of its spinal branch, it was permanently deprived of this property, by dividing the nervous twigs passing from the spine to the two ganglia, situated immediately above and below that, where the irritation was applied." 49.

### III. PATHOLOGY.

We know very little of this part of the subject. Neuralgia has been defined a "preternatural elevation of function in one or more of the sentient nerves, without corresponding excitement of the vascular, or of the great mass of the nervous system." This is, perhaps, as good a definition as can be formed. Its connexion with rheumatism is very close; but we cannot maintain that the two are identical. Many of the continental pathologists consider neuralgia as, in fact, neuritis of a chronic kind; but this doctrine is untenable. Inflammation, however chronic, soon induces alteration of structure; but it is well known that neuralgia may exist for years without effecting any perceptible change of structure in the nerve. It is useless to pursue this part of the subject farther.

### IV. TREATMENT.

There are few active remedies in the Pharmacopœia that have not been either proposed or tried for this terrible malady—a pretty clear proof of the intractable nature of the disease! Many remedies have acquired unmerited renown for a time, chiefly owing to the intermissions which naturally, and often capriciously occur in neuralgia—the post hoc ergo propter hoc argument being a very favourite one with most of our specific-mongers. Dr. R. justly observes that, "it is by a careful and patient investigation of the causes and habitudes of this dreadful malady, rather than by the introduction of a new remedy, that any improvement in the manner of treating it is to be hoped for." This observation applies indeed to every disease with which we have to contend. With the view of facilitating this investigation, Dr. R. divides neuralgia into four classes, according to the exciting causes.

"I.—Cases where the symptoms continue after the original cause has ceased to exist.

II.—Cases arising from functional disorders.

III.—Cases occasioned by causes of an irremediable nature.

IV.—Cases where the cause cannot be ascertained.

I.—Under the first class, are included those cases of Neuralgia, which can be traced to causes that have already disappeared; as when the pains continue after the subsidence of a cutaneous eruption, the removal of a tumour, the extraction of a tooth, &c. These cases generally yield rapidly to remedies which act powerfully on the nervous system; to be presently enumerated.

II.—In the cases comprehended under this section, the first indication is to remove the cause upon which the disease obviously depends; until this preliminary treatment has been accomplished, the remedies, which in the first class of cases, often afford relief, will generally be of no avail, and may increase the severity of the symptoms. But when the original disorder has been removed, the pains frequently disappear without further treatment; or at least, may now be removed by those remedies which had previously failed to make any impression on them.

III.—In this class are placed those unfortunate cases, depending upon causes of an irremediable character; but even in these, the nervous pains may be aggravated or calmed, according to the state of the disease with which they are connected.

The same remark is applicable to Neuralgia accompanying malignant diseases; the sufferings, even in these wretched cases, may be lightened, and sometimes rendered comparatively mild, by first subduing any inordinate cause of excitement of the diseased organ, and subsequently administering the more specific remedies for Neuralgia.

IV.—This section embraces those numerous cases, where the cause of the nervous pains cannot be discovered, and where the practice must therefore be in a great measure empirical." 70.

We shall not trouble our readers with a long list of remedies that have proved ineffectual or deceptious. Iron, arsenic, colchicum, iodine, calomel and opium, veratria, belladonna, purgation, counter-irritation—the insertion of morphia, strychnine, &c. under the skin, as in vaccination. Our author has tried this last method with some success. The section of nerves is now, we believe, almost entirely given up. Since Dr. Granville's book appeared we have applied the ammoniated counter-irritant with apparent benefit, to several facial neuralgiæ. It is a powerful application, but the heat and tingling soon subside, leaving the part more or less vesicated. Mr. Gardner, in Oxford-street will supply medical men with the antodyne, as directed by Dr. Granville; but the liquor ammoniæ fortis is just as good as the antodyne.

Our author, at page 73, goes from the topic of neuralgia generally to the specific forms, or rather seats of the disease, as the facial, cervical, cubito-digital, cutaneous, intercostal, ileo-scrotal, femora-popliteal, mammary, &c. which we pass over. He then takes up visceral neuralgia, in which he places angina pectoris. We coincide with him in opinion that change of structure in the heart itself is not essential to the symptoms of angina pectoris. Many dissections have shewn no organic affection; but, at the same time, we apprehend that, in a majority of cases, some structural lesion exists, although we may not be able to demonstrate it to the eye. We may even have found structural lesions, as of the valves, coronary arteries, or walls of the organ, which had little or no concern in the production of the phenomena during life. As was said before, the general opinion of the profession is now that angina pectoris is one of the most formidable of the neuralgiæ.

"If the principles which seem to determine the production of Neuralgic affections generally, be applied to the explanation of the symptoms of Angina Pectoris, they will lead to the following conclusions:—That many cases are to be ascribed to mechanical irritation of the cardiac nerves, by distention of the great vessels when exertion is made, or when the circulation is otherwise accelerated; whilst in others, the changes in the structure of the heart, are to be regarded as the means of determining the irritation excited by some other cause, to that organ, rather than as the origin of the disease." 129.

There is some obscurity in the latter part of the above passage. The treatment consists almost entirely in avoiding the lædentia—the exciting causes of the attacks. In the paroxysm, perfect quietude and stimulating anodynes must be employed.

Gastralgia is despatched by our author in a couple of pages, his chief object being to discriminate between gastralgia and gastritis—a discrimination not always easy, since the two states are not unfrequently combined in the same subject. The following passage contains the diagnosis of the two diseases.

"*Gastralgia.*—This affection is marked by paroxysms of pain, commonly described as lancinating, tearing, or burning, situated in the region of the stomach, and frequently extending to the thoracic parietes and to the back. It presents every degree of intensity, being sometimes merely a sensation of slight uneasiness, or of constriction in the epigastrium, whilst at others, the sufferings are of the most acute description. The duration of the attacks is also extremely various. In general, they do not continue longer than a few minutes, but are occasionally prolonged to several hours: they often terminate with a copious secretion of gas, either alone, or mixed with a quantity of limpid fluid, which rises spontaneously to the mouth, being sometimes insipid, but at others, having an acrid taste. Notwithstanding the disturbance in the principal organ of digestion, that process is seldom materially disturbed: the tongue generally re-remains clean, and the appetite good, and in some cases, it is voracious;—the bowels are mostly constipated. The pain is often relieved by food;—there is usually no thirst, or excitement of the pulse, or any other febrile symptom; and although the disease may have existed for many years, the patient often continues to bear the aspect of health.

The length of the intermission or remission, varies in different cases; two or more attacks, sometimes recur daily, at regular periods; in other cases, the intervals are much longer, and are irregular.

Gastralgia may be mistaken for chronic gastritis. By contrasting with the foregoing description, the following sketch of the latter affection, the diagnosis will be apparent, at least in common cases.

In Chronic Gastritis the pain is obtuse and confined to the epigastrium; it is increased by pressure, and aggravated by ingesta; it has no regular intermission; the tongue is commonly parched, and red at its tip and edges, with incrustation on its centre; the breath is fœtid; the mouth foul, there is much thirst, and a continual desire for cold drinks. The appetite is bad, and the sight of food disgusting, and when swallowed, it is almost instantly rejected. If it remain in the stomach, digestion is imperfectly performed, and is attended with acid or fœtid eructations, and febrile excitement.

The causes which act particularly in the excitement of gastralgia are, long abstinence, especially when the mind is anxious; an insufficient diet, with regard either to the quantity or quality of the aliment; uterine disturbance; irregular action of the heart, causing irritation of the pneumo-gastric nerves; pressure against the epigastrium, as required in the employment of certain artizans, such as shoemakers, weavers, &c." 133.

Our author has found no medicine more efficacious in gastralgia or enteralgia than the extract of nux vomica. It is a rough remedy to make free with.

We must pass over hepatalgia, nephralgia, cystalgia, and testalgia—the last of which has been so admirably described by Sir Astley Cooper. Hysteralgia has been well described by Gooch, Addison, Sir B. Brodie, and others. We suspect, from several cases that have occurred to us, that fæcal accumulations in the colon and rectum have kept up, or rather caused "irritable uterus," which carbonate of iron and tonics increased, rather than relieved. One of the most instructive cases which we remember to have witnessed, occurred lately, and was attended by Dr. Locock, Sir B. Brodie, and ourselves.

The patient was a young lady about 22 years of age, of pale complexion, and disposed to hysteria. She had been ailing, with various anomalous complaints, for more than a year—the chief phenomena being considered referable to irritable uterus, for which carbonate of iron had been given in large quantities, with various other tonics, stimulants, and anti-nervous medicines. Pains in the loins, hypogastrium, bladder, rectum, and uterus, were tormenting and rendered her life miserable. The bowels were habitually constipated. Dr. L. suspecting that accumulations existed in the bowels, examined the rectum, and found some hardened fæces there, which were removed by injections and aperients. Soon after this, however, all the symptoms became aggravated. The bearing-down pains exactly resembled labour pains, though the uterus was found to be perfectly healthy, and sickness and vomiting were added to the other phenomena. For days and nights she got no rest from the attacks, which came on every ten minutes or quarter of an hour, causing her to cry aloud. At this time we were requested to meet Dr. Locock, but as his engagements prevented him from meeting us, and as the friends of the young lady apprehended her death, we visited her, (by Dr. L.'s consent) alone. The uterus was found to be free from disease, but in the rectum we found, or thought we found, a large and very hard tumor, pressing on the uterus, and obstructing the rectum. We ordered enemata to be thrown up, and calomel to be given internally. Next day Sir B. Brodie examined the patient, and found the supposed tumor to be a mass of hardened fæces; and, in the course of that evening, a pound and a half of hard fæces, *chiefly composed of carbonate of iron*, were dug out of the rectum! It is hardly necessary to say that the young lady was instantly relieved from the most sleepless and excruciating agonies, and has since returned to the country in a state of improving health. The case is, we think, instructive, and may serve as a lesson to practitioners who are in the habit of prescribing large doses of carbonate of iron. Purgative medicines should always be given at short and regular intervals while the course of iron continues. Another caution regards the examination of the rectum, when the abovementioned symptoms present themselves. But, unfortunately, much mischief may be produced by the carbonate dropping into, and lurking in, the cells of the colon, where it is out of our reach.

The work concludes with a selection of twenty cases, in illustration of the disease in its various shapes and seats. The volume presents a fair epitome of our present knowledge of neuralgic affections.

